# Identification of *Brassica napus* small RNAs responsive to infection by a necrotrophic pathogen

**DOI:** 10.1186/s12870-021-03148-6

**Published:** 2021-08-11

**Authors:** Roshan Regmi, Toby E. Newman, Lars G. Kamphuis, Mark C. Derbyshire

**Affiliations:** 1 Centre for Crop and Disease Management, School of Molecular and Life Sciences, Curtin University, Bentley, WA 6102 Australia; 2 Commonwealth Scientific and Industrial Research Organisation, Agriculture and Food, Floreat, WA 6014 Australia

**Keywords:** Small RNA, micro RNA, Degradome, Fungal pathogen, *B. napus*, PHAS locus, TAS gene, Ta-siRNA, Pha-siRNA

## Abstract

**Background:**

Small RNAs are short non-coding RNAs that are key gene regulators controlling various biological processes in eukaryotes. Plants may regulate discrete sets of sRNAs in response to pathogen attack. *Sclerotinia sclerotiorum* is an economically important pathogen affecting hundreds of plant species, including the economically important oilseed *B. napus*. However, there are limited studies on how regulation of sRNAs occurs in the *S. sclerotiorum* and *B. napus* pathosystem.

**Results:**

We identified different classes of sRNAs from *B. napus* using high throughput sequencing of replicated mock and infected samples at 24 h post-inoculation (HPI). Overall, 3999 sRNA loci were highly expressed, of which 730 were significantly upregulated during infection. These 730 up-regulated sRNAs targeted 64 genes, including disease resistance proteins and transcriptional regulators. A total of 73 conserved miRNA families were identified in our dataset. Degradome sequencing identified 2124 cleaved mRNA products from these miRNAs from combined mock and infected samples. Among these, 50 genes were specific to infection. Altogether, 20 conserved miRNAs were differentially expressed and 8 transcripts were cleaved by the differentially expressed miRNAs miR159, miR5139, and miR390, suggesting they may have a role in the *S. sclerotiorum* response. A miR1885-triggered disease resistance gene-derived secondary sRNA locus was also identified and verified with degradome sequencing. We also found further evidence for silencing of a plant immunity related ethylene response factor gene by a novel sRNA using 5′-RACE and RT-qPCR.

**Conclusions:**

The findings in this study expand the framework for understanding the molecular mechanisms of the *S. sclerotiorum* and *B. napus* pathosystem at the sRNA level.

**Supplementary Information:**

The online version contains supplementary material available at 10.1186/s12870-021-03148-6.

## Background

Small RNAs (sRNAs) are short non-coding RNAs, ranging in size from 18 to 30 nucleotides (nt), that are important for gene expression regulation and genome stability in eukaryotes [1]. There are three major sRNA classes, microRNAs (miRNAs), short interfering RNAs (siRNAs) and P-element Induced WImpy (PIWI) associated RNAs (piRNAs); while the latter only occur in animals [2], the former two are found in plants. Different types of sRNAs have different biogenesis pathways [3].

sRNAs in plants silence gene expression through the RNA interference (RNAi) pathway. RNA dependent RNA polymerases (RdRps), Dicer-like proteins (DCLs), and Argonauts (AGOs) are the main RNAi pathway enzymes. RdRps facilitate the formation of dsRNAs, which are processed into sRNAs by DCLs. In general, AGO is thought to guide one of the strands (the guide strand) of dicer-processed sRNAs to silence complementary targets [4] while the other strand (the passenger ‘sRNA*’ strand), is often quickly degraded. However, recent studies have shown that the passenger strand can also have important gene silencing roles in plants [5–7]. In addition to complementary pairing with transcripts, siRNAs can also regulate gene expression epigenetically by RNA-directed methylation of complementary DNA [1].

Plants have many classes of siRNA [1, 8–10]. The main siRNA classes are the hairpin-siRNAs (hp-siRNAs), natural-antisense siRNAs (natsiRNAs), secondary siRNAs and heterochromatic siRNAs (hetsiRNAs) [1]. These classes have distinct biogenesis pathways and may involve DCL protein mediated cleavage of duplexes from either very long hairpins (hp-siRNAs) or double stranded RNAs generated from single stranded precursors by RdRp enzymes [1]. In addition to silencing mRNAs, miRNA-mediated cleavage of mRNAs or non-coding RNA precursors may also produce secondary siRNAs. These are often described as phased siRNAs (pha-siRNAs) as they appear at precise 21–22 nucleotide intervals from the miRNA cleavage site. Loci that produce pha-siRNAs are known as ‘PHAS’ loci [11]. The secondary RNAs produced by PHAS loci may silence the gene from which they are derived or they may act in *trans* to silence the expression of other genes; the latter type of secondary siRNAs are known as trans-acting siRNAs (ta-siRNAs) and their biogenesis loci are often referred to as ‘TAS’ genes.

While a large number of miRNA-triggered secondary siRNAs have been identified in the genomes of plants, only a few have been experimentally validated [12–15]. Four miRNA-triggered ta-siRNA families have been characterized in the model plant *A. thaliana* [13]. Of these, the miRNA390-triggered TAS3 genes were found to be conserved across various plant species. The formation of pha-siRNAs depends on several protein components, including SUPPRESSOR OF GENE SILENCING 3 (SGS3), RDR6 and DCL4 [13]. Studies on PHAS loci in different plants have shown that miRNAs trigger pha-siRNA production from many types of transcript, including noncoding RNAs, and the mRNAs of disease resistance and pentratricopetide repeat genes [16]. Nucleotide-binding site leucine-rich repeat (NBS-LRR) genes form the largest set of genes identified so far that can potentially produce pha-siRNAs upon binding of specific miRNAs [17].

Small RNAs in plants regulate genes associated with various biological processes such as seed germination [18], organ development [19] and maturation [20], signal transduction [21], and stress response [22]. Plants under pathogen attack may employ various sRNA-regulated immune pathways [23, 24]. For example, while studying the sRNAome in wheat cultivars during *Puccinia graminis* infection, Gupta et al. (2012) reported that miR408 exhibits different expression patterns in susceptible and resistant cultivars after a two-day course of infection [25]. Some immunity-related sRNAs have also been functionally characterised. For example, in the model plant *A. thaliana*, miR393 targets different auxin signalling genes to confer antibacterial resistance [22] and miR408 is a negative regulator of plantacyanins and laccase genes [26]. These latter genes have roles in stress responses, cell-to-cell signalling and maintaining plasticity and vigour of the cell wall. In addition, overexpression of miR7695 results in an incremental increase in resistance in rice against the blast fungus *Magnaporthe oryzae* [27]. During viral infection of plants, changes in the accumulation of miRNAs result in production of different pha-siRNAs [28]. In legumes and tomato, a number of miRNA families are involved in triggering pha-siRNAs by binding to the transcripts of NB-LRR genes [16, 29]. In tomato, the abundance of secondary siRNAs from disease resistance genes was lower during bacterial and viral infection, suggesting that pha-siRNA production is important for fine-tuning defence responses [29]. Recently Cui et al. (2020) demonstrated the role of miR1885-mediated ta-siRNA expression in maintaining plant growth and immunity in *B. napus* upon viral infection [30]. The roles of pha-siRNAs in plant response to bacterial and viral infection have been investigated in several studies but little is known about their roles in responding to pathogenic fungi. One of the few studies on this subject was by Wu et al. (2017). This study characterized pha-siRNAs produced by tomato in response to *Botrytis cinerea* infection [14]. It was found that many pathogen-responsive tomato pha-siRNAs downregulate transcription factors, which is suggestive of a broad role in the regulation of gene expression [15].

Canola (*B. napus*) is an economically important oilseed crop grown worldwide [31]. Sclerotinia stem rot (SSR), caused by the fungus *Sclerotinia sclerotiorum*, is an important disease that causes large economic losses in canola [32]. Some studies have been conducted in *Brassica* spp. to identify plant-specific miRNAs [33–35] under biotic and abiotic stresses. There have been two studies on *B. napus* miRNA expression upon *S. sclerotiorum* infection [36, 37]. However, these studies were performed with single sRNA libraries at 3, 12 and 48 h post-inoculation (HPI) without any replicates, which limits the proper understanding of differential expression of small RNAs in the *B. napus* response to *S. sclerotiorum*. Furthermore, in comparison to mature miRNAs deposited in miRBase for other plants like *M. truncatula*, *O. sativa* and *A. thaliana,* the number of miRNAs for *B. napus* is quite low, suggesting many miRNAs in *B. napus* are yet to be discovered. Finally, little is known about the triggers of PHAS loci in the *B. napus* genome and their functions in gene regulation in response to pathogens.

To assess differential expression of sRNAs, identify new pathogen-responsive miRNAs and characterise the role of secondary sRNAs in the *B. napus* response to *S. sclerotiorum*, we developed replicated sRNA libraries for mock-inoculated and *S. sclerotiorum* inoculated leaves 24 h post-inoculation to characterize different classes of sRNAs. To identify targets of these sRNAs, we also performed high throughput degradome sequencing and, for one gene, 5’RACE and RT-qPCR.

For the first time, we identified a large number of *B. napus* sRNAs up-regulated in response to *S. sclerotiorum* infection after 24 HPI. We also found evidence of pathogen-responsive activation of novel PHAS loci likely involved in regulation of disease resistance proteins. Our follow-up 5′-RACE and qPCR studies provided further evidence of sRNA-directed regulation of a gene involved in ethylene signalling.

## Results

### Overview of sequencing results

To determine the role of *B. napus* sRNAs during *S. sclerotiorum* infection we sequenced six sRNA libraries on the Illumina platform from three replicates each of mock and infected samples at 24 HPI when SSR symptoms manifested on leaves. A total of 152,090,773 raw reads were obtained from the six libraries. We retained 126,887,984 (83.24%) high quality reads after adapter trimming and length filtering (18–30 nt) from these six libraries (Table 1). Assignment and removal of ambiguous reads (that map to both plant and fungal genome) resulted in 41,797,278 unique *B. napus* sRNA reads that match best to the *B. napus* genome across all libraries. The reads that potentially originated from structural RNAs (rRNAs, snRNAs, snoRNAs) accounted for ~ 5% of this total. The clean, high-quality mappable reads were then aligned to the *B. napus* genome. The overall alignment rate was 88.7% with the highest percentage mapping in the mock samples (above 98%), while in infected samples an average of ~ 78.9% of reads mapped, ranging from 77 to 86.3% between replicates. Among the mapped reads, ~ 86% were mapped to more than one genomic locus revealing that these sRNAs originated from genomic repeats. From our dataset, we found 14% of sRNA reads that uniquely mapped to a single genomic locus. Table 1 provides an overall summary of the sequencing data.

**Table 1:** An overall summary of the sequencing data

Sample	Raw reads	Clean filtered reads^*^	Unique ***B. napus*** reads^**†**^	Structural RNA^**‡**^	Clean mappable reads^**††**^	Uniquely mapped reads	Reads mapping to multiple locations	Total mapped to ***B. napus***	Total mapped to ***B. napus*** (%)
Mock	23,754,736	20,144,362	8,944,885	318,393	8,626,492	1,047,207	7,416,613	8,463,820	98.1
Mock	22,377,118	18,983,632	7,767,860	311,538	7,456,322	1,103,293	6,242,937	7,346,230	98.5
Mock	31,509,749	26,430,696	11,421,524	450,137	10,971,387	1,535,228	9,301,292	10,836,520	98.8
Infected	26,483,807	20,626,924	3,779,014	202,125	3,576,889	445,577	2,310,074	2,755,651	77
Infected	23,182,766	18,818,235	3,866,998	223,567	3,643,431	462,630	2,214,409	2,677,039	73.5
Infected	24,782,597	21,884,135	6,016,997	508,508	5,508,489	570,565	4,180,632	4,751,197	86.3
Total	152,090,773	126,887,984	41,797,278	2,014,268	39,783,010	5,164,500	31,665,957	36,830,457	87.3

^*^Reads after size and adapter filtering

^†^Reads that mapped best to the *B. napus* genome from Bbsplit

^‡^Reads that potentially originated from structural RNAs (ribosomal RNA, snRNAs, e.t.c)

^††^Reads after removing potential structural RNAs that were used for ShortStack

**Fig. 1:**
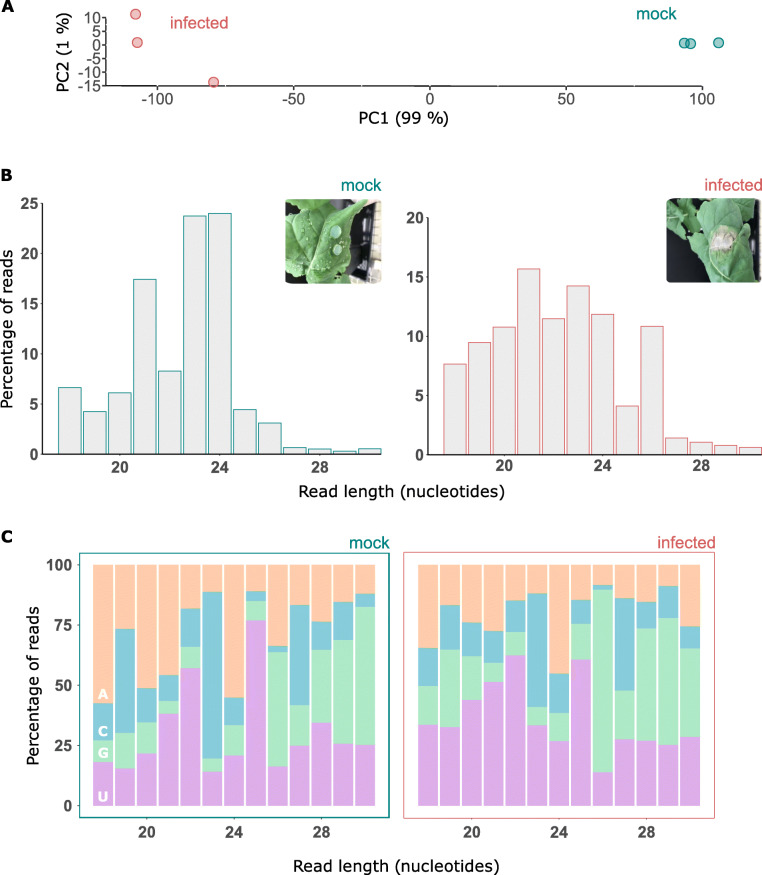
Changes in size class and 5′ nucleotide of *B. napus* sRNAs in response to *Sclerotinia sclerotiorum* infection. **A**A principal component analysis based on normalized read counts from DESeq2. The x axis shows principal component 1, which explained 99% of the variance, and the y axis shows principal component 2, which explained 1% of the variance. The infected samples are depicted with red circles and the mock samples depicted in turquoise. **B** Histogram of read sizes from pooled mock and infected samples. Inset: a representative picture of *B. napus* leaves under both treatments (24 h post-inoculation (HPI)). Left: for the pooled replicates of the mock sample, y axis depicts the percentage of reads across all three replicates and the x axis read length in nucleotides. Right: for the pooled replicates of the infected sample, same information as for the mock sample. **C** Results from pooled replicates for the mock (left) and infected (right). Showing percentage of reads (y axis) of each size class (x axis) that had each of the four nucleotides (AGCU) in their 5′ position

To determine the grouping of infected and mock libraries we performed a principal component analysis on mean normalized counts from DESeq2 (Fig. 1A). The principal component analysis showed the replicated datasets were well grouped for two treatment groups, i.e. mock and infected, suggesting large overall differences between these treatments. Mock and infected samples were separated along principal component 1, which explained 99% of the variance. There was some spread between the infected samples along principal component 2. However, variance between these samples along this axis is negligible, since only 1% of the variance was explained by PC2.

### Characteristic features of the *B. napus* small RNA population

To determine which sRNAs were induced in response to infection, we produced three sequencing replicates each from mock and infected samples. The following metrics are based on the pooled biological replicates for each treatment, mock and infected. Size class distribution and 5′ nucleotide bias are two important characteristics to determine the origin and activities of sRNAs. To determine whether there may be a difference in the composition of sRNA origins in mock and infected samples, we analysed the nucleotide length and 5′ nucleotide bias of these sRNAs. Interestingly, we found a difference in length distribution between mock and infected samples (Fig. 1B), suggesting that upon infection, sRNA biogenesis mechanisms are altered. In mock samples, almost 50% of total reads belonged to 24 and 23 nucleotide (nt) sRNAs followed by 21 nt. Adenine was enriched as the 5′ nucleotide in 24 nt sRNAs while cytosine was more abundant in 23 nt sRNAs. A 5′ nucleotide bias toward uracil was present mostly in 22 nt sRNAs (Fig. 1C).

In infected samples, size classes were more uniform than in mock samples (Fig. 1B). The most abundant read size was 21 nt with a slight 5′ uracil bias, followed by 23 nt with a slight cytosine bias (Fig. 1C). We also found a peak at 26 nt in infected samples with a 5′ guanine bias. Similarly, size classes of 18, 19, 20 and 22 nt were also more abundant in infected samples. However, among non-redundant reads in both samples, most were 24 nt. Apart from size distribution, the ratio of total to unique reads is also an important feature of an sRNA library [38]. The lower complexity of 21 nt sequences in the infected samples in comparison to 24-nt sequences indicates that a small number of unique reads of 21 nt are highly expressed while there are many different 24 nt sequences. Such features have been attributed to 24 nt heterochromatin sRNAs [39]. Small RNA size distributions for non-redundant reads in the mock and infected sample is shown in Supplementary Fig. 1.

Previous reports presented similar data with a 5′ uracil bias in 21 nt and a 5′ adenine bias in 24 nt sRNAs. The 24 nt 5′ adenine biased siRNAs have been previously shown to be involved in RNA dependent DNA methylation in *A. thaliana* with preferential loading into AGO4, while the 21 nt 5′ uracil biased sRNAs have preferential loading into AGO1 [40]. Overall, our results suggest a marked shift in the types of sRNAs expressed from mock to infected *B. napus* leaves.

### A total of 730 unique *B. napus* small RNAs are upregulated in response to *Sclerotinia sclerotiorum* infection

**Fig. 2:**
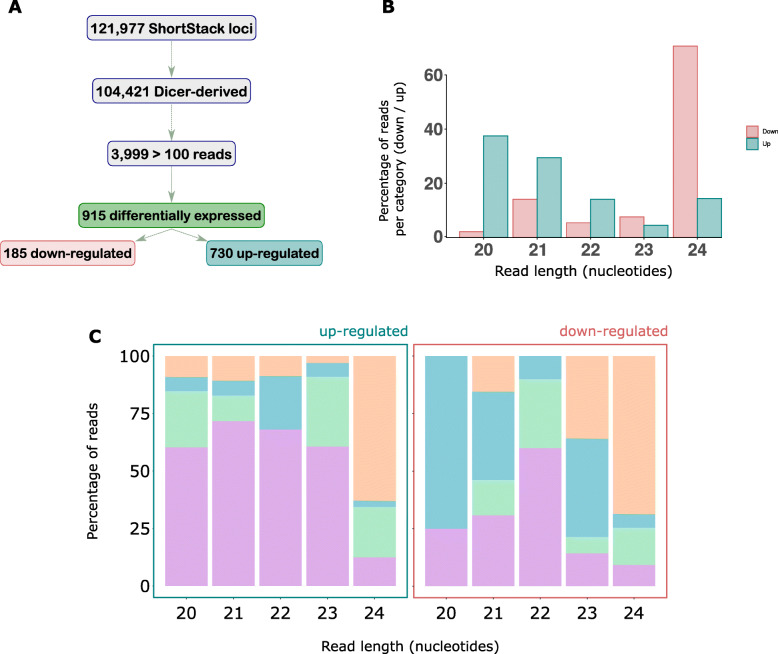
Small RNA population identified from *B. napus* genome in response to *Sclerotinia sclerotiorum* infection. **A** A flow chart showing total, Dicer-derived and highly expressed loci with a major RNA reads of = > 100 reads, as predicted by ShortStack, and differentially expressed loci identified from DESeq2. **B** Histogram of read sizes from upregulated and downregulated sRNA loci, y axis depicts the percentage of reads in each category (upregulated or down-regulated) and the x axis read length in nucleotides. **C** Histogram of read sizes showing percentage of reads (y axis) of each size class (x axis) that had each of the four nucleotides (AGCU) in their 5′ position for upregulated loci (left) and downregulated loci (right)

To assess what *B. napus* sRNAs accumulate in response to infection with *S. sclerotiorum*, we performed a differential expression analysis with DESeq2. We did not only consider differential expression of miRNAs but the entire sRNA-ome in *B. napus*. ShortStack predicted 121,977 sRNA loci, 104,421 of which were likely Dicer-derived. Among these loci, 3999 were highly expressed, with at least 100 raw major RNA sequencing reads (Supplementary Table 1). If these sRNAs were responding to infection, we hypothesised that they might be more expressed in infected samples as compared to mock samples. We found 915 loci significantly altered in their expression in infected samples compared to the mock samples. Among these, 730 were upregulated in *B*. napus after *S. sclerotiorum* infection; these loci produced 565 unique sRNAs based on the major sRNAs predicted by ShortStack (Fig. 2A).

These 730 upregulated sRNAs were mostly enriched for 20 and 21 nt sequences, while the 185 downregulated major sRNAs were enriched for 24 nt sequences (Fig. 2B). Uracil was enriched at the 5′ ends of all the size classes for upregulated sRNAs except 24 nt, which had a 5′ adenine bias (Fig. 2C). However, downregulated sRNAs exhibited a 5′ cytosine bias at 20 nt and 21 nt (Fig. 2C). Size classes 22 and 24 nt shared a common 5′ bias of uracil and adenine respectively in both sample groups. Overall, our data add weight to the hypothesis that sRNA classes with distinct biogenesis and targeting pathways were expressed in response to *S. sclerotiorum* challenge.

### Stress responsive genes are targeted by up-regulated small RNAs

**Fig. 3:**
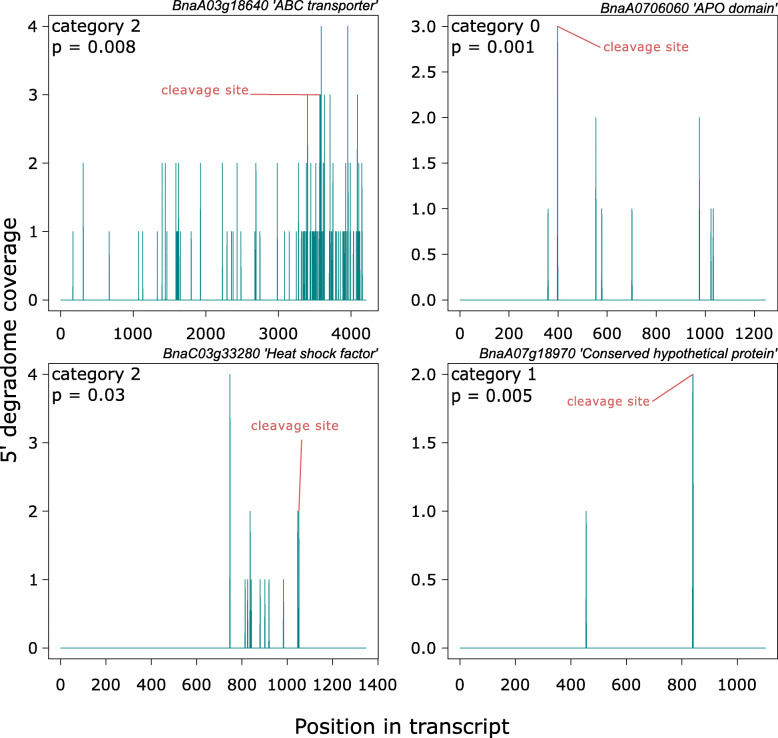
Representative Target plots (T-plots) for infection-specific targets of upregulated sRNAs. The x-axis shows the transcript position in the target genes and the y-axis shows the 5′ read coverage at different positions; cleavage sites predicted by PARESnip2 are labeled in red. The category and *p* value given by PARESnip are shown in the top left hand corners of graphs and the gene IDs and their putative functions are shown above

We used the degradome sequencing data to investigate targets of sRNAs upregulated during infection. A total of 64 target genes were identified from upregulated sRNAs this way (Table 2). Representative T-plots for four of these genes that were identified in infected libraries, which were assigned to different PARESnip2 confidence categories, are presented in Fig. 3. Among these 64 targets, 10 were found in both libraries, resulting in 29 and 15 unique targets for mock and infected samples, respectively. Genes that were possibly regulated by small RNAs up-regulated during infection were annotated with transcription factor-related InterPro terms such as ‘ABC transcription factor’, ‘heat shock response’, ‘disease resistance protein-like’, ‘Zinc finger l domain’ and ‘leucine zipper domain’. This suggests that *B. napus* transcriptional regulatory networks may be modified by sRNAs specifically induced during infection.

**Table 2:** Predicted degraded *B. napus* targets of upregulated *B. napus* small RNAs based on degradome sequencing data analysed using PARESnip2

Gene ID	Category^*^	Cleavage position	Alignment score	Duplex MFE^**ƚ**^	Perfect MFE	MFE Ratio	***p***-value^**‡**^	Library^**ƚƚ**^
BnaA08g20190D	3	1211	3.5	−23.6	−32.9	0.717325	0.019978	M
BnaA02g03600D	0	748	4	−20.6	−28.9	0.712803	0.000756	M, I
BnaC03g43110D	2	219	4	−24.2	−32.3	0.749226	0.039024	M
BnaC05g00280D	2	1144	3	−24.6	−29.6	0.831081	0.016924	M
BnaA10g11740D	3	955	4	−25.8	−35.2	0.732955	0.032497	M
BnaA01g22910D	3	848	4	−28.4	−39.6	0.717172	0.038079	M
BnaCnng67150D	3	68	3	−31	−37.9	0.817942	0.01312	M
BnaA08g28020D	3	917	3.5	−29.1	−40.8	0.713235	0.033058	M
BnaA07g08860D	2	1153	1	−32.6	−39.2	0.831633	0.002904	M
BnaA09g27990D	2	1156	1	−32.6	−39.2	0.831633	0.002897	M,I
BnaC05g21250D	2	1162	1	−32.6	−39.2	0.831633	0.002885	M,I
BnaC07g11360D	2	1141	1	−32.6	−39.2	0.831633	0.002929	M
BnaA03g36860D	3	336	4	−29.6	−41.5	0.713253	0.039506	M
BnaA06g36560D	1	345	2	−33.8	−41.5	0.814458	0.008645	M
BnaC07g17320D	3	1383	2	−33.8	−41.5	0.814458	0.008237	M
BnaA04g18170D	3	388	4	−33.7	−42.3	0.79669	0.025798	M
BnaA10g18410D	3	1347	4	−31.8	−39.7	0.801008	0.021698	M
BnaA02g06410D	2	627	4	−27.5	−39.2	0.701531	0.009812	M
BnaA04g07950D	0	2282	3.5	−31.4	−43.5	0.721839	0.001638	M,I
BnaA07g25390D	0	2174	3.5	−31.4	−43.5	0.721839	0.001718	M,I
BnaA08g17390D	0	2282	4	−30.6	−43.5	0.703448	0.003145	M,I
BnaA09g26170D	0	2333	4	−30.6	−43.5	0.703448	0.003069	M,I
BnaC03g59640D	2	2267	4	−30.6	−43.5	0.703448	0.015732	M
BnaC05g23210D	0	2324	4	−30.6	−43.5	0.703448	0.00308	M,I
BnaC06g27170D	0	2168	3.5	−31.4	−43.5	0.721839	0.001722	M,I
BnaCnng25410D	0	2267	3.5	−31.4	−43.5	0.721839	0.001648	M,I
BnaC01g07210D	3	37	4	−33.7	−43.2	0.780093	0.030433	M
BnaAnng05290D	3	792	4	−26.4	−34.4	0.767442	0.009756	M
BnaA06g11000D	3	299	3	−27.3	−33.2	0.822289	0.042121	M
BnaA06g11010D	3	299	3	−27.3	−33.2	0.822289	0.042404	M
BnaC04g20940D	3	215	0	−33.2	−33.2	1	0.005071	M
BnaC08g38450D	1	1481	3.5	−27.1	−37.5	0.722667	0.001168	M
BnaCnng05480D	2	1980	3.5	−24.2	−34.3	0.705539	0.020979	M
BnaC07g37000D	2	487	0.5	−35.5	−35.9	0.988858	0.003279	M
BnaA09g16090D	3	3478	3.5	−28.4	−35.2	0.806818	0.037582	M
BnaA04g26610D	2	5713	4	−23.8	−33.9	0.702065	0.01421	M
BnaC04g50670D	2	5713	4	−23.8	−33.9	0.702065	0.013297	M
BnaC01g18190D	2	466	3	−22.1	−31	0.712903	0.013158	M
BnaCnng58300D	3	891	4	−26.5	−34.4	0.770349	0.018106	M
BnaA02g03600D	1	748	4	−20.6	−28.9	0.712803	0.003782	I,M
BnaC03g33280D	3	1046	3	−24.3	−28.8	0.84375	0.002259	I
BnaC05g38210D	2	80	4	−25.5	−35.3	0.72238	0.003571	I
BnaA07g18970D	1	840	3.5	−34.6	−46.4	0.74569	0.002773	I
BnaA07g26110D	2	1696	4	−30.9	−39.8	0.776382	0.036546	I
BnaA09g27990D	2	1156	1	−32.6	−39.2	0.831633	0.00145	I,M
BnaC05g21250D	2	1162	1	−32.6	−39.2	0.831633	0.001444	I,M
BnaA07g06060D	0	398	3.5	−31.5	−40.6	0.775862	0.000816	I
BnaA04g07950D	0	2282	3.5	− 31.4	−43.5	0.721839	0.001638	I,M
BnaA07g25390D	0	2174	3.5	−31.4	−43.5	0.721839	0.001718	I,M
BnaA08g17390D	0	2282	4	−30.6	−43.5	0.703448	0.002753	I,M
BnaA09g26170D	0	2333	4	−30.6	−43.5	0.703448	0.002686	I,M
BnaC05g23210D	0	2324	4	−30.6	−43.5	0.703448	0.002695	I,M
BnaC06g27170D	0	2168	3.5	−31.4	−43.5	0.721839	0.001722	I,M
BnaCnng25410D	0	2267	3.5	−31.4	−43.5	0.721839	0.001648	I,M
BnaC03g30850D	3	1850	3.5	−23.1	−29.2	0.791096	0.035487	I
BnaA05g25860D	3	743	4	−20.8	−29.5	0.705085	0.017563	I
BnaA09g37590D	2	3003	4	−24.1	−33	0.730303	0.049431	I
BnaA09g04340D	3	45	4	−25.9	−33.3	0.777778	0.01457	I
BnaA08g00270D	3	152	4	−28	−36	0.777778	0.021834	I
BnaAnng16680D	2	735	3.5	−34.1	−46.4	0.734914	0.016373	I
BnaA06g04910D	3	2188	4	−24	−30.4	0.789474	0.011989	I
BnaA03g18640D	2	3633	2.5	−25.5	−30.4	0.838816	0.001911	I
BnaC08g21330D	3	587	4	−26.8	−37.1	0.722372	0.013281	I
BnaC02g30040D	2	367	2	−27.8	−34.3	0.810496	0.007331	I

^*^Category derived from PARESnip2 based on rules of Falgren and Carrington, 2010

^ƚ^*MFE* minimum free energy

^‡^P value based on randomisation test implemented in PARESnip2

^ƚƚ^Library in which this tag was detected; *M* mock library, *I* infected library

### Identification of conserved miRNAs in *B. napus*

**Fig. 4:**
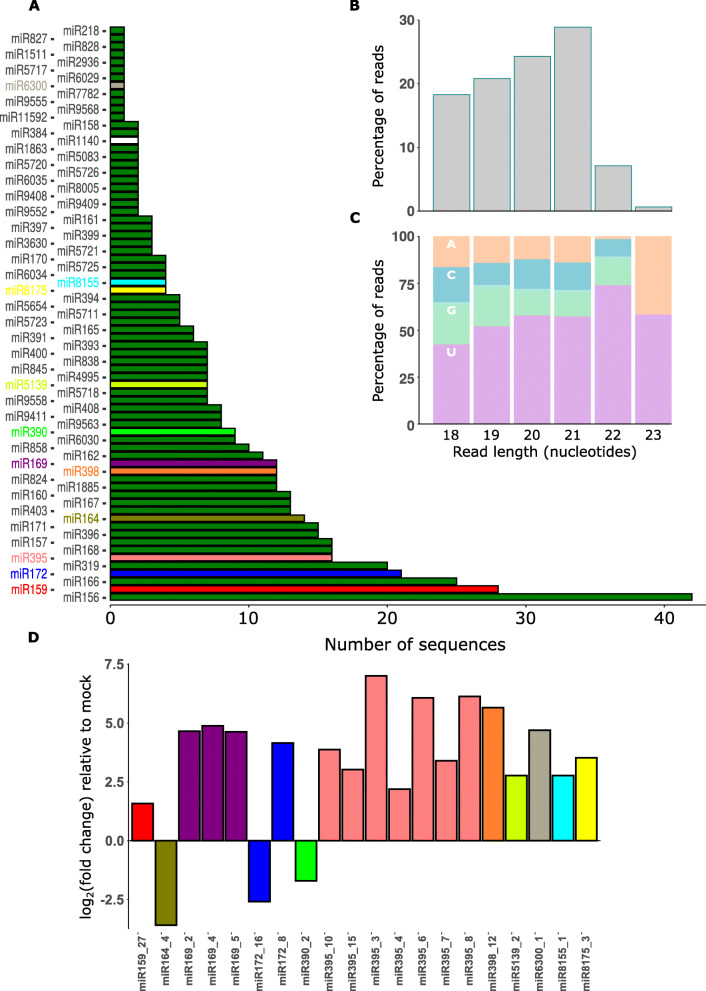
Prediction of infection-responsive microRNAs from the *B. napus* genome. **A** Histogram of the 73 conserved miRNA families. The y-axis shows the identified conserved miRNA family name and the x-axis shows the number of sequences (isomiRs) identified for each miRNA family; red bars are the significantly differentially expressed miRNA families. An isomiR is one of a family of highly similar miRNA sequences derived from either the guide or passenger strand. Green miRNA families were not differentially expressed. Those in other colours contained differentially expressed miRNAs and the colours correspond with **D**. **B** Histogram of read sizes from 529 conserved miRNAs. The y axis depicts the percentage of reads and the x axis read length in nucleotides. **C** Histogram of the 529 miRNAs showing percentage of reads (y axis) of each size class (x axis) that had each of the four nucleotides (AGCU) in their 5′ position. **D** 20 differentially expressed miRNAs with log_2_(fold change) on the y axis. Colours correspond with miRNA families in **A**

Several miRNAs are evolutionarily conserved in the plant kingdom [41]. We assessed whether conserved *B. napus* miRNAs were expressed during *S. sclerotiorum* infection. Therefore, all six clean libraries were searched against miRBase (Release 22.1). From our libraries, we identified 73 conserved miRNA families with 529 mature miRNA sequences. We found that 61 miRNA families had more than one sequence while 12 miRNA families had only one mature sequence predicted (Fig. 4A). Among these miRNA families, miR156 had 42 isomiR sequences followed by miR159, and miR166 with 28 and 25 sequences, respectively. Most of these miRNA sequences were 21 nt long, followed by 20, 19 and 18 nt (Fig. 4B). There was a 5′ uracil bias in 18–22 nt long miRNA sequences, which agreed with previously reported results in miRNA studies in different plant species (Fig. 4C). Using degradome sequencing, we found that from the 73 conserved miRNA families, 718 and 1406 cleaved products (Supplementary Table 2) were obtained from infected and mock libraries, respectively. Four levels of degradome cleavage site confidence, based on read mapping characteristics, are described in [42, 43]. Category 0 is the most confident, followed by categories 1, 2 and 3. Among the 718 cleavage events in the infected sample, 507 were in category 0, followed by category 2, 3, and 1 with 90, 87, and 34 events, respectively, based on the abundance of fragment transcripts in the library. Thus, most of the conserved miRNA targets identified with degradome sequencing were of relatively high confidence [44]. Several target genes were likely silenced by more than one miRNA family. Among these target genes, 158 non-redundant transcripts were found in infected samples. Among the 158 targets in infected samples, miR160, miR164, miR167, and miR396 were predicted to target more than 10 genes each. Similarly, miR156, miR6030, miR400, miR393, miR172 and miR171 were predicted to target eight genes (Supplementary Table 3). We found an additional 43 conserved miRNA families compared with *B. napus* miRNAs recorded in miRbase. These miRNAs were reported previously in several studies to regulate gene expression in plants during biotic and abiotic stress [45–48].

### Pathogen responsive miRNAs cleave plant immune response genes in the infected sample

To determine whether any degradation of transcripts was specific to the infected samples, we filtered out all the genes from the infected sample that were also targeted in the mock sample, resulting in 50 targets (Supplementary Table 4). Altogether, 172 Interpro domains were found in these genes. These genes had functions such as transcriptional regulation, disease resistance, and posttranscriptional gene silencing. We found several miRNAs that were shown to have a role in plant and pathogen interactions from this set also.

### Seventeen miRNAs belonging to 9 miRNA families were significantly upregulated during infection

We performed differential expression analysis on the individual conserved miRNAs with raw read counts from the miRprof analysis. Among the conserved miRNAs, only 20 miRNAs were differentially expressed (Fig. 4D; Supplementary Table 5). Among these, seventeen miRNAs belonging to 9 miRNA families were upregulated during infection and three miRNAs were downregulated. Among these upregulated 9 miRNA families, miR395 had 7 isomiRs, miR169 had 3 isomiRs while miR159, miR172, miR398, miR6300, miR8155, miR8175, and miR5139 had one isomiR each. The up-regulated miRNA sequences exhibited log_2_(fold change) values of between 1.58 and 6.13. A total of 16 of the 17 miRNAs had a log_2_(fold change) of more than 2.

The three downregulated miRNAs belonged to the miRNA families miR164, miR72, and miR390. These miRNAs exhibited log_2_(fold change) values during infection of between − 1.7 and − 3.59; two exhibited log_2_(fold change) values below − 2. Interestingly, miR172 had two isomiRs with different expression patterns, with one upregulated and the another downregulated during infection.

Among the 17 upregulated miRNAs, we found 5 genes potentially cleaved by a member of the miR159 family, miR159_27 (log_2_(fold change) = 1.56; P-adjusted = 0.034), and a member of the miR5139 family, miR5139_2 (log_2_(fold change) = 2.77; P-adjusted = 0.0042), in the infected samples. Surprisingly, we found 3 transcripts cleaved by downregulated miR390 member, miRNA390_2 (log_2_(fold change) = − 2.94; P-adjusted = 0.04), in the infected sample, while there was no cleavage of these genes in the mock samples.

### RNA structure-aided prediction algorithms identify 135 novel *B. napus* micro RNA loci

After filtering out the exact matches of conserved miRNAs to miRBase, 135 novel miRNA producing loci that did not have any hits in miRBase were identified from the *B. napus* genome. Among these miRNAs, 67 loci were found to have both passenger strand (miRNA* or ‘star’) and mature strand reads revealing the confidence of these novel miRNAs as per annotation criteria. A detailed description of the novel miRNA loci is given in Supplementary Table 6**.**

From our study, we did not find any cleavage signal from the novel miRNAs predicted from miRDeep2. We used the same set of miRNAs to predict targets using the psRNA target server. From psRNA target, 12,104 genes were putatively targeted by these miRNAs. Several psRNA targets might be false positives since it is entirely based on a theoretical in silico procedure, whilst a degradome signal is a better reflection of the biological cleavage. It remains to be confirmed whether these novel miRNAs have genuine targets or not.

### Nine *B. napus* PHAS loci are differentially expressed in response to *Sclerotinia sclerotiorum* infection

PHAS loci have not been very well characterised in *B. napus*. Therefore, we aimed to identify expressed PHAS loci in the *B. napus* genome from our sequencing data set. We found 26 PHAS loci in the *B. napus* genome. The genes associated with predicted PHAS loci were annotated by aligning PHAS locus sequences to the NCBI Nucleotide Collection (nr/nt). Among the 26 PHAS genes, about half were related to disease resistance proteins (5 genes), non-coding RNAs (5 genes), and chloroplast related (3 genes). In addition, single genes were found for metal tolerance, pentatricopeptide repeat, cop9 signalosome complex subunit, and photosystem II protein D1. Nine PHAS genes were not homologous to any sequences in NCBI. A total of 182 pha-siRNAs were produced from these loci. Among these siRNAs, 41 were highly expressed, with a read abundance of more than 100 reads.

Since miRNAs are key triggers of pha-siRNA expression, we used the psRNA target server to find the cleavage sites in PHAS loci from the conserved miRNAs we identified. We found six PHAS loci potentially triggered by conserved miRNAs (Table 3). All excised PHAS clusters with their corresponding pha-siRNAs are shown in Supplementary File 1. The miR390-triggered PHAS gene *TAS3* was found to be conserved across different species. In this study, we found two genes possibly targeted by miR390, one of which had sequence similarity to *TAS3* in *A. thaliana*.

**Table 3:** An overview of the characteristics of PHAS loci identified using PHAS tank

PHAS locus ID	Length	Phased ratio	Phased abundance	Phased number	Phased score	Triggering miRNA^**a**^	Description of BLAST hit	Differential expression
chrCnn_random_466	791	0.398	3845	16	52.538		Uncharacterized	
chrA04_130	917	0.448	274	19	47.821	miR4995, miR6035	chloroplast	
chrC01_614	331	0.568	3417	7	32.332		Uncharacterized	Upregulated
chrA03_random_16	434	0.6	630	8	30.96		Disease resistance protein	Upregulated
chrA01_443	245	0.79	877	5	26.769	miR390	non coding RNA	Downregulated
chrUnn_random_22	621	0.468	159	11	26.078		chloroplast	
chrC09_754	621	0.468	159	11	26.078		chloroplast	
chrA01_random_47	245	0.769	877	5	26.05		non coding RNA	Downregulated
chrA07_478	287	0.621	823	6	25.004		metal tolerance protein	
chrA01_532	371	0.666	111	7	21.953		Disease resistance protein	Upregulated
chrC09_1169	392	0.747	1217	4	21.216	miR838	Uncharacterized	
chrA02_413	455	0.381	2189	7	20.533		Uncharacterized	
chrAnn_random_753	308	0.531	547	6	20.073	miR390	non coding RNA	
chrA09_223	476	0.314	579	10	19.96	miR1885	Disease resistance protein^b^	
chrC05_811	392	0.393	559	8	19.894	miR838	non coding RNA	
chrA01_533	329	0.746	288	4	16.888		Disease resistance protein	Upregulated
chrC02_807	371	0.668	109	5	15.662		Disease resistance protein	
chrAnn_random_716	413	0.7	182	4	14.562		Uncharacterized	
chrA10_253	329	0.376	198	7	13.935		Photosystem II protein D1	Upregulated
chrC01_385	266	0.444	237	5	12.145		non coding RNA	
chrC09_1045	266	0.4	152	5	10.041		COP9 signalosome complex subunit	Downregulated
chrC09_1044	434	0.373	788	4	9.949		Pentatricopeptide repeat	
chrCnn_random_695	391	0.35	234	5	9.558		Uncharacterized	
chrA04_52	413	0.359	206	5	9.558		Uncharacterized	
chrC08_352	392	0.46	118	4	8.772		Uncharacterized	Downregulated
chrC07_411	350	0.305	175	4	6.294		Uncharacterized	

^a^Based on psRNA target comparison of conserved miRNA sequences with all PHAS loci

^b^Evidence for cleavage from degradome sequencing

**Fig. 5:**
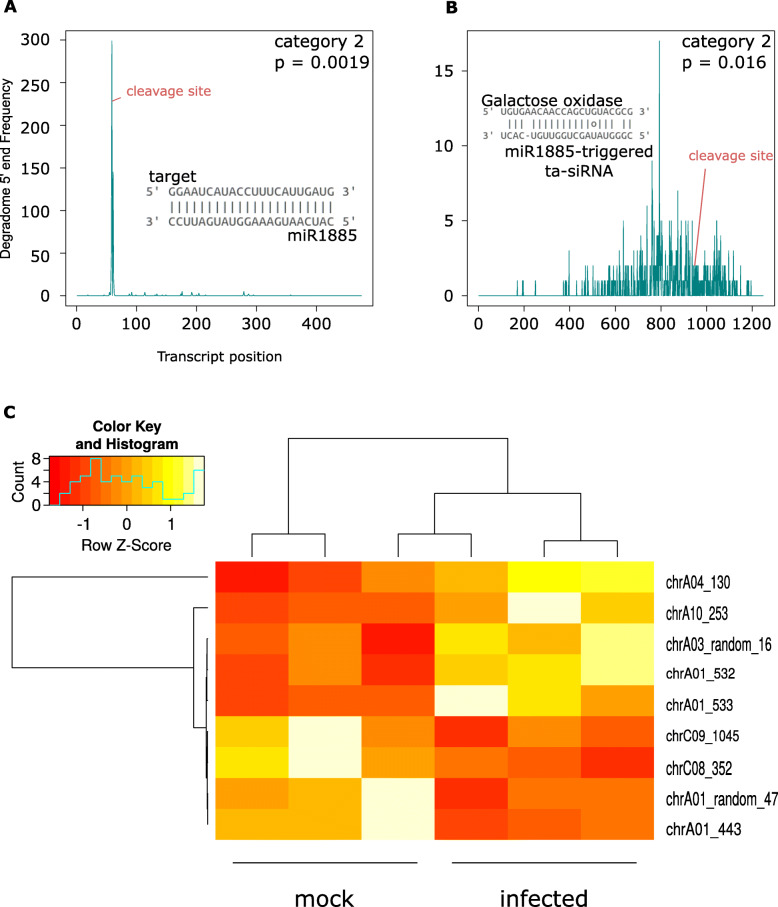
Degradome validation of a TAS gene triggered by miRNA 1885. **A** Target plot (T-plot) of the cleavage site on a TAS gene putatively targeted by miRNA 1885. The transcript position in the target gene is on the x-axis and the y-axis shows the 5′ read coverage at each position; the cleavage site predicted by PARESnip2 is labelled in red. The category and *p* value of the PARESnip2 test are given in the top left hand corner of the graph. **B** T-plot of cleavage site on a galactose oxidase gene putatively targeted by one of the ta-siRNAs produced by the miRNA 1885-triggered TAS gene. The x-axis shows the transcript position in the target gene and the y-axis shows read 5′ coverage at each position; the cleavage site predicted by PARESnip2 is labelled in red. The category and *p* value from PARESnip2 are given in the top right hand corner of the graph. **C** A heat map of 9 differentially expressed PHAS loci plotted with normalized counts from DESEq2

We were able to confirm likely cleavage of one of these loci during infection using degradome sequencing (category 2, *P* = 0.0019; Fig. 5A). We did not find a degradome signal for another five miRNA-triggered loci predicted with the psRNA target server. The locus we were able to validate was likely targeted by the conserved miRNA miR1885. Recently, it has been shown experimentally that miR1885 plays a key role in targeting PHAS loci residing within NBS-LRR genes to trigger ta-siRNA production [30]. Accordingly, we found that this locus had homology to NBS-LRR proteins. We identified 10 likely ta-siRNAs produced from this PHAS locus. From the degradome signal, only one of these targets BnaC05g49720D, a galactose oxidase, beta-propeller, had a cleavage signal from degradome sequencing (category 2, *P* = 0.016; Fig. 5B) in the infected sample. Possibly, *B. napus* miRNAs regulate gene expression in response to *S. sclerotiorum* infection through the production of miRNA triggered ta-siRNAs.

Differential expression analysis of PHAS loci showed five loci were upregulated and four were downregulated during infection. Among upregulated loci, three were related to disease resistance with a log2fold change ranging from 0.78 to 1.87. The remaining two genes were chloroplast and photosystem II protein D1 with a log2fold change of 0.57 and 1.73, respectively. Among downregulated loci, two loci were non-coding RNAs with a log2fold change of − 0.7 and − 0.96, one was related to COP9 signalosome complex subunit (log2fold change = (− 0.82) and the remaining one was not characterized. Figure 5C shows a heat map of 9 differentially expressed PHAS loci.

To gain a global overview of genes targeted by pha-siRNAs we used the psRNA target server to find the targets of 41 highly expressed pha-siRNAs. We found 5918 transcripts that might be regulated by this class of sRNA. We did GO term enrichment analysis of these targets and found regulation of several biological processes (Supplementary Table 7). The terms ‘posttranscriptional gene silencing’ (GO:0035194), ‘cellular potassium ion homeostasis’ (GO:0030007), ‘regulation of ARF protein signal transduction’ (GO:0032012) and ‘threonyl-tRNA amino acylation’ (GO:0006435), ‘oxidation-reduction process’ (GO:0055114), and ‘regulation of transcription DNA-templated’ (GO:0006355), ‘carbohydrate metabolic process’ (GO:0005975)’ were significantly enriched.

We also specifically investigated the targets of the miR1885-triggered ta-siRNAs. We found 1601 targets of these sRNAs with psRNA target. GO term enrichment analysis showed that these ta-siRNAs possibly regulate protein phosphorylation (GO: 0006468), transcription factors (GO: 0045944), vesicle mediated transport proteins (GO:0016192), and fucose metabolic pathway genes (GO:0006004) (Supplementary Table 8).

### Further analysis of sRNA targeting using 5′ rapid amplification of cDNA ends and quantitative PCR

**Fig. 6:**
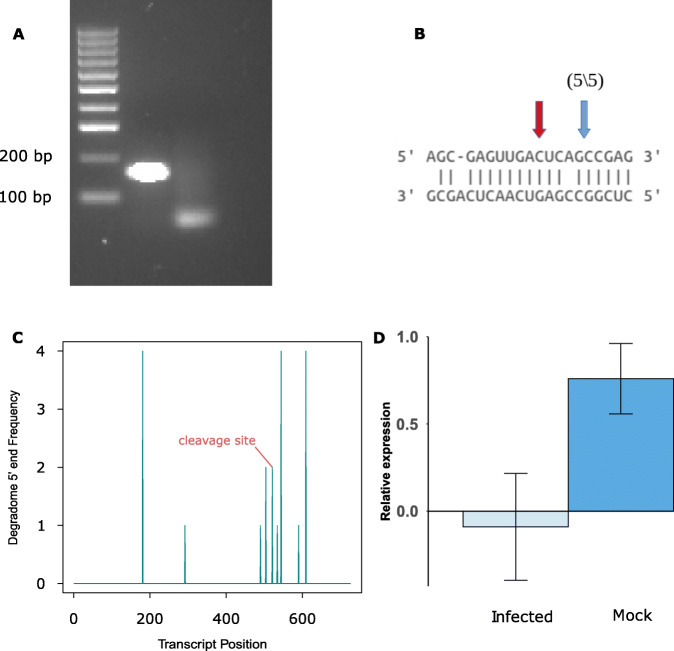
5′ Rapid amplification of cDNA ends (RACE), degradome result, and qPCR for an ethylene response factor gene putatively cleaved by a novel sRNA. **A** Gel electrophoresis of the 5′-RACE result showing a band of the correct size; the second lane is a no template control. The full gel image is in Supplementary Fig. 1. **B** Sequence complementarity of the sRNA and its target. The blue arrow shows the cleavage site identified from sequencing the 5′-RACE product and the red arrow shows the cleavage site identified with degradome sequencing. **C** Target-plot (T-plot) of the degradome result of the 5′-RACE validated gene showing the transcript position in the target gene (x-axis) and read 5′ coverage at each position (y-axis); the cleavage site predicted by PARESnip2 is labelled in red. **D** RT-qPCR result of target gene in mock and infected sample (x-axis) and relative expression of gene to the house keeping actin gene

5′ RACE was used to find putative cleavage sites in the novel sRNA target gene *BnaA01g27570D*, which is an ethylene response factor. This gene was chosen as ethylene signalling has a well-documented role in plant immunity to pathogens [42] and, although ethylene response factors have a demonstrated role in response to *S. sclerotiorum* [49], there is little understanding of how they might be regulated by sRNAs. This novel siRNA expressed from chromosome A01 is 22 nt long, not conserved or characterised, is neither a pha-siRNA nor a miRNA. Figure 6A shows the 5′-RACE product of the predicted cleavage site from the infected sample; the full gel image is in Supplementary Fig. 2. A T-plot of the cleavage signal is shown in Fig. 6 C. We also assessed the expression of this target gene during infection using RT-qPCR. The average log (2^-∆Ct^) value calculated for mock and infected sample was 0.76 and − 0.09 respectively with a standard deviation of 0.35 and 0.53 Fig. 6C. The combined degradome, 5’RACE, and qPCR results showed that a novel *B. napus* sRNA likely regulates an ethylene response factor gene during *S. sclerotiorum* infection, leading to a decrease in its expression. Overall, the integrated degradome, 5’RACE, and RT-qPCR results showed the regulation of a plant immune response gene by a novel siRNA.

## Discussion

Small RNA-omics studies have revealed the tight regulation of host immune pathways in plants [23, 50, 51]. In our study, we identified different classes of sRNAs in *B. napus* plants that responded to infection with *S. sclerotiorum* and showed how they may be involved in regulating different sets of genes using degradome sequencing.

We found evidence of sRNA-mediated regulation of an ethylene response factor gene (*BnaA01g27570D*), which we further investigated using RT-qPCR and 5′-RACE. The likely cleavage site identified through degradome sequencing and 5′-RACE and concomitant reduction in expression of this gene during infection suggest that it has a role in the plant response to pathogen attack. Enhanced ethylene production is an early response of plants to perception of pathogen attack, leading to induction of defence systems [52]. In *B. napus*, ethylene responsive element binding factors have been predicted to control biological processes related to defence signalling, secondary metabolite production and redox regulation. The closest homologue of *BnaA01g27570D* in *A. thaliana*, RAP2.3, has been shown to co-localise in the nucleus with another ethylene response factor, ORA59, to mediate defence to the bacterial pathogen *P. carotovorum* [53]. Since RAP2.3 has a known positive role in disease resistance and ethylene is generally found to be a positive contributor to defence, it is intriguing that our study showed a reduction in expression of *BnaA01g27570D* caused by potential sRNA-mediated regulation during infection. Several hypotheses could be put forward to explain this. For example, the pathogen could elicit responses in the plant that dampen ethylene-responsive immunity. Alternatively, some components of the ethylene response system could negatively regulate immunity, as shown in some other systems [42]. Further studies are required to determine the biological significance of sRNA cleavage of this particular ethylene response factor upon *S. sclerotiorum* infection of *B. napus*.

The distribution of size classes of total sRNAs was different between mock and infected samples. The size class of sRNAs gives insights into their biogenesis, for example 21 nt sRNAs are processed by DCL1 and DCL4, whereas 22 nt sRNAs are formed through the action of DCL2; 24 and 26 nt sRNAs are formed by DCL3 [54]. Similarly, sRNAs with 5′ uracil, adenine or cytosine are loaded into AGO1, AGO2 and AGO4 or AGO5, respectively [55]. Furthermore, it has been reported previously that variation in sRNA lengths also has effects on downstream function of sRNAs [56]. In the mock sample, both redundant and non-redundant distributions peaked at 24 nt. However, in the infected sample, the redundant distribution had a major peak at 21 nt and the non-redundant distribution had peak at 24 nt. Overall, our data suggest that a different set of sRNA biogenesis pathways is initiated upon infection with *S. sclerotiorum*.

A total of 730 sRNA loci were upregulated in *B. napus* in response to *S. sclerotiorum* infection. The upregulated sRNAs mostly belonged to the size classes of 20 and 21 nt with a 5′ bias of uracil, whereas downregulated sRNAs were overrepresented for 24 nt sequences with varied 5′ biases. This suggests that upon pathogen infection, the host recruits different DCL and AGO proteins for subsequent gene silencing. The upregulated sRNAs likely silenced many genes related to stress signaling, as determined from the degradome data. This suggests that the enhancement of disease symptoms might be accompanied with the negative regulation of plant immune genes during *S. sclerotiorum* infection of *B. napus*.

The miRNAs are sRNAs which are produced from short hairpin precursors. Based on these criteria, thousands of miRNAs have been identified and deposited in miRBase from many plants. The majority of these miRNAs show high conservation. However, only 92 mature *B. napus* miRNAs have been discovered so far. These are less numerous than those of *A. thaliana* (428), *M. truncatula* (756), and *O. sativa* (738) revealing several miRNAs are yet to be discovered in this species.

Two previous studies have assessed *S. sclerotiorum* responsive *B. napus* miRNAs at time points 3, 12, and 48 HPI [36, 37]. A total of 227 [36] and 77 [37] conserved miRNA sequences were identified in these studies. Both of these studies were conducted with a single sRNA sequencing library per sample. The microarray miRNA expression analysis resulted in detection of 68 infection-responsive miRNAs, [36] and 10 of these were further analyzed with stem loop qPCR. Similarly, 10 miRNAs were found to be differentially expressed in Jian et al. [37], while only one miR166 was found to be commonly differentially expressed with respect to Cao et al. study [36]. Here, we found 529 mature conserved miRNAs belonging to 73 miRNA families, along with 20 infection-induced miRNAs based on a replicated differential expression analysis. The enhanced number of miRNAs in our study might be due to the replicated dataset and increased sequencing depth. We did not find any common differentially expressed miRNAs in comparison to the two previous studies [36, 37]. This might be due to the different time points*, B. napus* variety, tissue collection and *S. sclerotiorum* strains we used*.* Nevertheless, the degradome data suggested these miRNAs regulate expression of transcription factors related to development and defense responses, which corroborate previous findings.

Moreover, 135 novel miRNA loci were discovered with 67 loci that had both mature and star (passenger strand) read counts. Although this adds to the overall number of *B. napus* miRNAs identified, we were not able to identify any likely targets of these miRNAs. This suggests that they either do not have targets or that they regulate genes through a non-cleavage mechanism such as inhibition of translation [43]..

Expression of a large number of transcription factors and auxin signaling pathway genes was likely regulated by these identified conserved miRNAs based on the infected sample degradome data. Some of these miRNAs had multiple targets in specific classes. We found 50 unique cleaved products from the infected sample that were not present in the mock samples. These genes were related to transcription factors, disease resistance proteins, and posttranscriptional silencing. For example, miR824, miR390/miR5083, miR403/miR838, miR5139, miR1885, cleaved transcripts of genes containing leucine rich repeats, zinc finger transcription factors, protein kinases, and disease resistance protein-encoding genes, respectively. Similarly, miR166 and miR858 cleaved transcripts of homeodomain and sant/myb domain-containing genes, respectively. MiR824 was shown previously in the Brassicaceae to have a role in the heat stress response [57]. It is worth mentioning here miR858 has been shown to negatively regulate MYB transcription factors, thereby controlling resistance to pathogen infection in *A. thaliana* [58]. Similarly, plant homeodomain proteins, which are potentially regulated by miR166 in this study, control transcriptional regulation of pathogen defense-related genes [59] . These findings suggest that miRNAs are involved in regulation of multiple aspects of the immune response of *B. napus* to *S. sclerotiorum*.

From our study, we found 20 infection-induced miRNAs. These miRNAs were previously shown to have roles in stress responses in different pathosystems [50, 60–62]. However, we only found degradome evidence of cleavage of the transcripts of five genes, *BnaA03g22590D*, *BnaA05g27620D*, *BnaCnng31260D*, *BnaCnng49390D*, and *BnaA02g31560D*, which were targeted by the upregulated miRNAs miR159_27 and miR5139_2 in the infected samples. Four of these five genes were potentially cleaved by miR159_27 and the remaining gene by miR5139_2. These genes contained Interpro domains such as SANT/myb domain, homeodomain, and zinc finger domain. Surprisingly, miR390_2, whose expression was ~ 69% (log_2_(0.31) = − 1.7) lower in the infected sample had three target genes, whose mRNAs were cleaved only in the infected samples (*BnaA06g09370D*, *BnaC05g49670D*, *BnaC08g16190D*). It suggests that these genes were not expressed in the mock sample.

On the other hand, we found 11 genes whose transcripts were cleaved in the mock sample from different isomiRs of upregulated miR395, while we did not find any of these cleaved genes in the infected samples. These data suggest that gene regulation by sRNAs is quite complex and the cleavage events are not always dependent on the expression pools and other variables might also come in play.

MiR390 has been shown to have a role in the formation of ta-siRNAs and regulate auxin response factor genes [63] while miR395 plays an important role in sulphur assimilation [64]. MiR159 is present in the majority of land plants where it regulates genes encoding R2R3 MYB domain transcription factors that transduce the gibberlin (GA) signal [65] and have roles in growth, development, and biotic stress responses. It was shown recently that cotton and *Arabidopsis* plants accumulate increased levels of miR159 in response to the fungus *Verticillium dahliae* [66]. miR159 was also reported to have a role in *Arabidopsis* root galls under the attack of root knot nematodes, since lines lacking the miR159-Gibberlic acid MYB pathway had better resistance to root knot nematode. Moreover, the miR159-GA MYB pathway has been shown in *A. thaliana* to promote the programmed cell death response [67]. Our finding of four MYB domain genes with transcripts cleaved by miR159 suggested that miR159 has a role in *B. napus* responses to *S. sclerotiorum*. However, further investigation will be needed to understand the precise role of this pathway.

In previous studies, miR5139 was shown to be regulated by ethylene in petal growth and was first detected in perennial herbs [68] but no specific functions were allocated to this miRNA. Later it was also shown to have tissue-specific expression in wheat [69]. However, no homolog of miR5139 was reported in *B. napus* to date. Here, we found the expression level of miR5139 was nearly 7 times higher (log_2_(6.96) = 2.8) during infection and it was found to cleave the transcript of the gene *BnaA02g31560D* which encodes a zinc finger domain-containing gene. Zinc finger domains are reported to be present in plant resistance related proteins that are involved in effector triggered immune responses [70]. Our data shows that under the influence of *S. sclerotiorum* attack, *B. napus* deploys miR5139 to negatively regulate Zinc finger domain-containing genes as a defence response strategy [70].

Although we did not find any degradome cleavage signals for the other differentially expressed miRNAs, there were some interesting miRNAs identified here which were shown previously to have a role in plant responses against pathogens. miR164 was shown to manipulate programmed cell death in *A. thalina.* Overexpression of miR164 target genes enhanced disease symptoms [71]. miR169 was found to negatively regulate rice immunity genes during infection by the rice blast fungus [50]. miR6300 and miR8175 were highly upregulated in *Alternaria*-treated tomato plants compared to the control with a log_2_(fold change) of 3.13 or 2.65. Here, we found a 3.5 and 4.7 fold increase of these two miRNAs after *S. sclerotiorum* infection [61].

With the aid of the degradome library we showed that miR1885 can trigger a disease resistance *TAS* gene which subsequently produces 10 ta-siRNAs for gene regulation. This particular miRNA was recently shown to directly silence the *B. napus* TIR-NBS-LRR resistance gene *BraTNL1* and the TAS gene *BraTIR1*, cleavage of which generates sRNAs that regulate the photosynthesis-related gene *BraCP24* [30]. By regulating both immunity and basal growth, this miRNA may be essential for optimizing resource allocation during development. This miRNA has been shown to be expressed at a low abundance under most conditions apart from flowering time, when the plant requires synergistic reductions in the levels of phytosynthesis and pathogen response. In our study, we did not observe a change in expression of miR1885 in response to *S. sclerotiorum* infection, although we observed potential cleavage of a galactose oxidase, beta propeller protein-encoding transcript (*BnaC05g49720D*) by one of the small RNAs derived from the TIR-NBS-LRR TAS gene it likely activates. However, such proteins have quite varied roles [72, 73], so it is not possible to come to any conclusions on the biological significance of this observation. Identification and elucidation of the regulatory network of pha-siRNAs is important so that the expression of these pha-siRNAs can be controlled by changing the expression of their miRNA triggers. This strategy could be useful to modulate the degree of silencing of endogenous and exogenous target genes.

## Conclusions

In conclusion, our comprehensive data set allowed us to investigate overall pathogen-responsive RNA interference-based regulation of host transcripts and the actions of specific small RNA classes in response to pathogen attack. Our data suggest that targets of infection induced sRNAs including miRNAs may be associated with stress response genes. *B. napus* plants may differentially express both pha-siRNAs and conserved miRNAs when challenged with a necrotrophic pathogen. An integrated analysis revealed that a *B. napus* sRNA regulates an ethylene response factor gene during pathogen attack. Ethylene response factors regulate several jasmonate (JA) and (ET) pathways and are key players in plant innate immunity [51]. Our combined degradome, 5′ RACE and qPCR results showed that the expression of one of the ethylene response genes is suppressed in *B. napus* after *S. sclerotiorum* infection. The silencing of this gene was mediated by a novel sRNA which was not characterized before.

## Methods

### Biological materials

An Australian *S .sclerotiorum* isolate (CU8.24) originally collected from South Stirling WA was used for infection assays [74]. Mature sclerotia were cut into halves and placed onto 9 cm Petri dishes containing potato dextrose agar (PDA). After germination from the sclerotium, mycelium was subcultured onto fresh PDA medium. After 48 h of incubation, mycelial plugs were placed on fully expanded second or third leaves of one-month-old *B. napus* plants (AV Garnet). Seeds of AV-Garnet were originally acquired from The Australian Grains GeneBank (accession AGG95718BRAS1). The plants were grown for a month in a growth chamber with 16 h of daylight and 8 h of darkness. After infection, plants were carefully covered with a polythene bag to increase the humidity, thereby facilitating the infection process. Twenty-four HPI, a characteristic necrotic lesion was observed on the infected leaves. The infected tissues were carefully excised using sterilized scissors and immediately frozen in liquid nitrogen and stored at − 80 °C until RNA extraction for sequencing. For mock samples, PDA only agar plugs were used without any fungal mycelium. Three leaves from three different plants were pooled together for each replicate. For small RNA sequencing, three biological replicates were sequenced separately while 2 degradome libraries were sequenced by pooling all infected replicates as one library and all mock replicates as another library.

### Total RNA extraction and sequencing

Total RNAs were extracted using the TRIZOL reagent following the manufacturer’s protocol (Invitrogen Carlsbad, CA, USA). After extraction, total RNAs were quantified using a Nanodrop spectrophotometer, and Qubit. The integrity of RNA samples was checked using agarose gel electrophoresis. Three to five μg and 25–30 μg of total RNA were sent to Novogene (Singapore) for small RNA and degradome sequencing respectively. The sRNA sequencing was done using the NEBNext® Multiplex Small RNA Library Prep Kit for Illumina® with single end 50 bp reads according to the manufacturer’s protocol. Degradome sequencing was done as mentioned in [75]. In brief, the construction of a degradome library was started from the degradation site (with monophosphate group) of the degraded mRNA. The sequencing adaptors were added to both ends of the degradation library and a library size of around 200–400 bp was selected. The sequencing was performed on a Hiseq 2500 SE50.

### Analysis of small RNA sequencing data

Raw reads were trimmed using cutadapt software (version 1.15) optimized for single-end reads with a setting of cutadapt -a (universal True Seq adapter) -m18 -M30 [76]. The quality of filtered reads was checked by running in FastQC [77]. Reads with a length in the range of 18–30 nt were retained. Trimmed infected reads were assigned to the fungal [78] and plant [79] reference sequences using bbsplit software in the bbmap [80] program keeping ambig2 option set as toss. The option ambig2 removes all the reads that map to both references with equal confidence. The reads that were unique to the *B. napus* genome were kept for prediction of *B. napus* sRNAs.

For prediction of *B. napus* sRNA biogenesis loci, clean reads were aligned to the reference genome of *B. napus* allowing for a maximum of two mismatches. We used ShortStack [81] to gain an overall idea of sRNA producing loci from the *B. napus* genome and to characterize highly expressed sRNA loci after infection. Each library was used as a single entity without collapsing for input into ShortStack. For conserved miRNA prediction, we matched the clean reads against miRBase (version 22) (http://www.mirbase.org) using the miRProf program in UEA small RNA workbench [82]. The reads that matched to mature miRNAs in the miRbase database with 0 mismatches were considered as conserved miRNAs. The remaining reads that did not match miRBase were parsed for the prediction of novel miRNA-producing loci using the miRDeep2 program [83]. Differential expression analysis was done using the Bioconductor package DESeq2 in R with estimate variance – mean dependencies. We used the raw cluster abundance of 6 individual libraries from ShortStack to find differentially expressed sRNA loci. The sRNAs with a Benjamini-Hochberg corrected *p*-value of < 0.05 were considered as differentially expressed sRNAs [84]. For the differential expression analysis of conserved miRNAs, we used the raw counts for the individual miRNA sequences identified from the miRprof program.

The phasing patterns of loci were predicted with a Perl script from the PHAS tank software (version 1.0) [85]. To find the miRNA triggered phased initiator loci, complementary cleavage sites of predicted miRNAs on PHAS loci were searched using the psRNA target server assuming that the 10th nucleotide position on the miRNA is a cleavage start position of its targeted PHAS loci [15].

### Analysis of sRNA targeting using in silico target prediction and degradome sequencing data

To determine whether reads originated either from the plant or the fungus we used bbsplit [80] to categorise infected degradome reads as fungal or plant-specific reads. The filtered reads were separated from potential structural RNAs by filtering against the RFAM database [86] using the program Infernal (version 1.1.3) [87].

We used either the psRNA target server with a default setting and an expectation score of 5 for computational prediction of sRNA targets [88] or PARESnip2 [89] to validate the cleavage sites from Degradome datasets following the rules of Fahlgren and Carrington [90]. We retained the targets with category number 0–3. Category-0 are targets with a degraded products having degradome peaks more than one read and the maximum on the transcript where there is only one maximum. Category-1 are those having degradome peaks greater than one read and are the maximum on the transcript, but there is more than one maximum. Category-2 peaks are those that have reads more than one and are above the average fragment abundance on the transcript. Category-3 signals are those that have greater than one read and are below or equal to the average fragment abundance on the transcript. Further verification of PHAS locus activation by specific miRNAs was also investigated using the degradome sequencing tags with PARESnip2. To gain a more detailed understanding of silencing by different sRNA classes, we used four different datasets: the one highly expressed major RNA per locus from the ShortStack program, the conserved miRNAs from miRbase, novel miRNAs annotated from miRDeep2 and phasiRNA identified by PHAS tank.

### Gene ontology enrichment analysis

Gene ontology enrichment analysis was conducted on sRNA target transcripts with the topGO program from R 3.6.1 Bioconductor package. GO term enrichment tests were performed separately on mock and infected samples. In each case, the background set was all GO terms in the *B. napus* genome and the foreground set was any gene with evidence of degradome targeting. The mock and infected samples were compared to identify genes that were enriched in the mock and depleted in the infected sample or vice versa. GO terms with a *p*-value < 0.05 were considered as significantly enriched or depleted [91].

### Five prime rapid amplification of cDNA ends of a cleaved target

We conducted a 5′-RACE experiment on one of the ethylene response factor genes from our degradome dataset that is potentially cleaved by a plant sRNA. The reason for choosing this gene is directed by previous pieces of literature where these classes of genes were shown to be crucial for defence responses in plants against pathogen attack [92] and high confidence complementary site between this siRNA and the target gene according to psRNA target server. Furthermore, the sRNA targeting this gene was hitherto uncharacterised, and it is not a miRNA or phasiRNA. We used two independent samples collected from independent infection assays to conduct 5′ RACE using the first choice RACE kit following the manufacturer’s protocol (Applied Biosystems, USA) without adding calf intestinal Phosphatase enzyme. One sample was the same as the one used for degradome sequencing while the other was not.

In brief, 5′ RACE adapters were ligated to 5 μg of total RNA, which was reverse transcribed using the universal RT primer provided in the kit and the MMLV transcriptase. The first PCR was conducted on 1 μL of cDNA with a 5′ outer RACE primer and gene-specific outer primer. The second nested PCR was done using the first PCR product with a 5′ inner RACE primer and inner nested PCR primers. The PCR product was visualized on a 2% Agarose gel. The amplified DNA fragment was gel purified and cloned into TOP TA vector and 5 independent clones were Sanger sequenced.

### Quantitative polymerase chain reaction

The expression levels of a sRNA target gene were analysed by RT-qPCR. One to five ug of total RNAs from mock and infected *B. napus* leaf samples were converted to cDNA using the MMLV reverse transcriptase kit (Sigma-Aldrich). The cDNA samples were then diluted 1/20 before qPCR. The qPCR analysis was performed using the Bio-Rad Taq Universal SYBR Green Supermix according to the manufacturer’s instructions. The thermocycler settings were 95 °C for 2 min, then 95 °C for 15 s, 60 °C for 30 s and 72 °C for 15 s, and cycled for 40 times, followed by 72 °C for 2 min. Three biological and three technical replicates were used for each sample. Relative expression was calculated as per log (2^-∆Ct^) method normalized to the *B. napus* housekeeping actin gene. The primers and adapters used for 5′ RACE and qPCR experiments are listed in Supplementary Table 9.

## Supplementary Information

**Additional file 1: Supplementary File 1**. Output of the software PHAS Tank showing candidate PHAS loci.

**Additional file 2: Supplementary Fig. 1**. Size distribution of unique sRNAs.

**Additional file 3: Supplementary Fig. 2**. The full image of the cropped gel appearing in Fig. 6.

**Additional file 4: Supplementary Table 1**. All small RNA biogenesis loci identified using the program Sho:rtStack.

**Additional file 5: Supplementary Table 2**. Cleaved products of the 73 miRNA families. All targets identified using degradome sequencing in infected and mock samples are included.

**Additional file 6: Supplementary Table 3**. Targets of conserved microRNAs based on degradome sequencing data from the infected sample. Table includes genes targeted by multiple miRNAs and multiple genes targeted by the same miRNA. Targets only found in the infected sample are included.

**Additional file 7: Supplementary Table 4**. Genes targeted by conserved miRNAs only in the infected sample.

**Additional file 8: Supplementary Table 5**. Conserved micro RNA sequences with evidence of differential expression during infection.

**Additional file 9: Supplementary Table 6**. Known and novel miRNAs identified in this study.

**Additional file 10: Supplementary Table 7**. GO term enrichment analysis of 5918 transcripts possibly regulated by pha-siRNAs based on psRNA target analysis.

**Additional file 11: Supplementary Table 8**. GO term enrichment analysis of psRNA target-predicted targets of 1601 targets of miR1885-triggered ta-siRNAs.

**Additional file 12: Supplementary Table 9**. Primers used in this study.

## Data Availability

The smallRNA and degradome sequencing data has been deposited in GenBank under BioProject PRJNA678586.

## Abbreviations

ta-siRNA: Trans-acting short interfering RNA
siRNA: Short interfering RNA
sRNA: Small RNA
pha-siRNA: Phased short interfering RNA
miRNA: Micro-RNA
DCL: Dicer-like
natsiRNA: Natural antisense short interfering RNA
hetsiRNA: Heterochromatic short interfering RNA
SSR: Sclerotinia stem rot
RDR: RNA dependent RNA polymerase
RISC: RNA-induced silencing complex
NBS-LRR: Nucleotide binding site leucine-rich repeat
RACE: Rapid amplification of cDNA ends
qPCR: Quantitative PCR
nt: Nucleotide
GO: Gene ontology
