# Correction to: Identification of *Brassica napus* small RNAs responsive to infection by a necrotrophic pathogen

**DOI:** 10.1186/s12870-021-03178-0

**Published:** 2021-08-26

**Authors:** Roshan Regmi, Toby E. Newman, Lars G. Kamphuis, Mark C. Derbyshire

**Affiliations:** 1 Centre for Crop and Disease Management, School of Molecular and Life Sciences, Curtin University, Bentley, WA 6102 Australia; 2 Commonwealth Scientific and Industrial Research Organisation Agriculture and Food, Floreat, WA 6014 Australia

**Correction to: BMC Plant Biol 21, 366 (2021)**

**https://doi.org/10.1186/s12870-021-03148-6**

Following publication of the original article [1], the author has found two mistakes in the title made by the BMC production office. The title of the original article and this erratum have been changed from ‘fIdentification of *B. napus* small RNAs responsive to infection by a necrotrophic pathogen’ to ‘Identification of *Brassica napus* small RNAs responsive to infection by a necrotrophic pathogen.

The original article has been corrected.
