# Supplementary figures and images for: Identification of Brassica napus small RNAs responsive to infection by a necrotrophic pathogen
Source: BMC Plant Biol. 2021 Aug 11;21:366. doi: 10.1186/s12870-021-03148-6 (PMC8356391; doi:10.1186/s12870-021-03148-6)

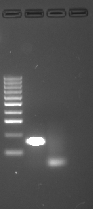

Supplement: Supplementary file 3 — Additional file 3: Supplementary Fig. 2. The full image of the cropped gel appearing in Fig. 6. [file 12870_2021_3148_MOESM3_ESM.png]
